# Supplementary material for: Clinical Burkholderia pseudomallei isolates from north Queensland carry diverse bimABm genes that are associated with central nervous system disease and are phylogenomically distinct from other Australian strains
Source: PLoS Negl Trop Dis. 2022 Jun 14;16(6):e0009482. doi: 10.1371/journal.pntd.0009482 (PMC9236262; doi:10.1371/journal.pntd.0009482)
Supplement: S1 Text — Table A in S1 Text. Known sequence multi-locus types identified in Queensland B. pseudomallei isolates. Table B in S1 Text. Novel Queensland B. pseudomallei multi-locus sequence types, ~ flags the closest known allele. Table C in S1 Text. Details of the Burkholderia pseudomallei reference data used in this study. (DOCX) [file pntd.0009482.s001.docx]

**S1 Text – Supplementary tables**

**Table A:** Known sequence multi-locus types identified in Queensland *B. pseudomallei* isolates.

| **ST** | **No. identified** |
| --- | --- |
| 24 | 2 |
| 35 | 1 |
| 70 | 5 |
| 109 | 1 |
| 151 | 1 |
| 235 | 1 |
| 252 | 3 |
| 254 | 1 |
| 257 | 2 |
| 283 | 2 |
| 286 | 1 |
| 591 | 2 |
| 593 | 2 |
| 594 | 1 |
| 958 | 1 |
| 1041 | 1 |
| 1042 | 1 |
| 1667 | 2 |
| 1667 | 1 |
| 1707 | 1 |
| 1711 | 1 |
| 1712 | 1 |
| 1754 | 1 |
| 1756 | 1 |
| 1757 | 1 |
| 1760 | 1 |
| 1769 | 1 |

| **Isolate** | **ST** | **ace** | **gltB** | **gmhD** | **lepA** | **lipA** | **narK** | **ndh** |
| --- | --- | --- | --- | --- | --- | --- | --- | --- |
| CAM_103 | - | ace(1) | gltB(4) | gmhD(13) | lepA(2) | lipA(1) | narK(24) | ndh(1) |
| CAM106 | - | ace(1) | gltB(~32) | gmhD(13) | lepA(4) | lipA(6) | narK(12) | ndh(11) |
| CAM126 | - | ace(1) | gltB(6) | gmhD(3) | lepA(19) | lipA(1) | narK(3) | ndh(1) |
| CAM157 | - | ace(1) | gltB(1) | gmhD(24) | lepA(4) | lipA(1) | narK(6) | ndh(1) |
| CAM162 | - | ace(1) | gltB(2) | gmhD(3) | lepA(39) | lipA(6) | narK(19) | ndh(1) |
| CAM179 | - | ace(1) | gltB(2) | gmhD(3) | lepA(~33) | lipA(1) | narK(6) | ndh(1) |
| CAM181 | - | ace(1) | gltB(2) | gmhD(3) | lepA(39) | lipA(6) | narK(19) | ndh(1) |
| CAM188 | - | ace(1) | gltB(6) | gmhD(3) | lepA(19) | lipA(1) | narK(3) | ndh(1) |
| CAM196 | - | ace(1) | gltB(2) | gmhD(3) | lepA(~33) | lipA(1) | narK(6) | ndh(1) |
| CAM30 | - | ace(1) | gltB(2) | gmhD(13) | lepA(2) | lipA(20) | narK(~124) | ndh(1) |
| CAM47 | - | ace(8) | gltB(2) | gmhD(22) | lepA(2) | lipA(18) | narK(26) | ndh(1) |
| CAM71 | - | ace(1) | gltB(7) | gmhD(13) | lepA(2) | lipA(5) | narK(3) | ndh(1) |
| CAM84 | - | ace(1) | gltB(2) | gmhD(3) | lepA(39) | lipA(6) | narK(19) | ndh(1) |
| CAM87 | - | ace(1) | gltB(4) | gmhD(6) | lepA(2) | lipA(15) | narK(3) | ndh(1) |
| TSV101 | - | ace(1) | gltB(28) | gmhD(6) | lepA(4) | lipA(1) | narK(3) | ndh(~13) |
| TSV103 | - | ace(1) | gltB(2) | gmhD(23) | lepA(2) | lipA(1) | narK(38) | ndh(11) |
| TSV11 | - | ace(8) | gltB(2) | gmhD(3) | lepA(2) | lipA(3) | narK(38) | ndh(~1) |
| TSV121 | - | ace(1) | gltB(23) | gmhD(13) | lepA(2) | lipA(3) | narK(~124) | ndh(1) |
| TSV130 | - | ace(8) | gltB(4) | gmhD(13) | lepA(2) | lipA(6) | narK(~6) | ndh(11) |
| TSV136 | - | ace(1) | gltB(15) | gmhD(13) | lepA(2) | lipA(1) | narK(33) | ndh(1) |
| TSV138 | - | ace(23) | gltB(4) | gmhD(13) | lepA(~33) | lipA(8) | narK(19) | ndh(1) |
| TSV141 | - | ace(1) | gltB(2) | gmhD(22) | lepA(22) | lipA(3) | narK(19) | ndh(11) |
| TSV146 | - | ace(1) | gltB(2) | gmhD(6) | lepA(2) | lipA(1) | narK(6) | ndh(4) |
| TSV152 | - | ace(1) | gltB(23) | gmhD(~27) | lepA(2) | lipA(1) | narK(2) | ndh(1) |
| TSV164 | - | ace(8) | gltB(2) | gmhD(22) | lepA(4) | lipA(4) | narK(~23) | ndh(1) |
| TSV179 | - | ace(12) | gltB(15) | gmhD(3) | lepA(4) | lipA(15) | narK(12) | ndh(1) |
| TSV185 | - | ace(1) | gltB(4) | gmhD(23) | lepA(36) | lipA(8) | narK(44) | ndh(1) |
| TSV188 | - | ace(1) | gltB(~4) | gmhD(14) | lepA(2) | lipA(1) | narK(22) | ndh(4) |
| TSV203 | - | ace(1) | gltB(1) | gmhD(13) | lepA(4) | lipA(6) | narK(47) | ndh(11) |
| TSV207 | - | ace(1) | gltB(2) | gmhD(23) | lepA(19) | lipA(6) | narK(22) | ndh(11) |
| TSV20 | - | ace(1) | gltB(4) | gmhD(13) | lepA(2) | lipA(2) | narK(19) | ndh(11) |
| TSV24 | - | ace(1) | gltB(1) | gmhD(13) | lepA(4) | lipA(6) | narK(8) | ndh(7) |
| TSV286 | - | ace(1) | gltB(1) | gmhD(3) | lepA(4) | lipA(1) | narK(~2) | ndh(1) |
| TSV39 | - | ace(1) | gltB(23) | gmhD(13) | lepA(4) | lipA(1) | narK(2) | ndh(1) |
| TSV41 | - | ace(1) | gltB(15) | gmhD(3) | lepA(2) | lipA(6) | narK(50) | ndh(1) |
| TSV52 | - | ace(23) | gltB(2) | gmhD(6) | lepA(2) | lipA(6) | narK(6) | ndh(4) |
| TSV69 | - | ace(15) | gltB(2) | gmhD(24) | lepA(2) | lipA(6) | narK(6) | ndh(11) |
| TSV92 | - | ace(8) | gltB(15) | gmhD(13) | lepA(2) | lipA(3) | narK(12) | ndh(15) |

**Table B.** Novel Queensland *B. pseudomallei* multi-locus sequence types, ~ flags the closest known allele.

**Table C.** Details of the *Burkholderia pseudomallei* reference data used in this study.

| Strain | Accession | URL |
| --- | --- | --- |
| TSV 48 | GCA_000770495.1 | ftp://ftp.ncbi.nlm.nih.gov/genomes/all/GCA/000/770/495/GCA_000770495.1_ASM77049v1 |
| TSV202 | GCA_000770565.1 | ftp://ftp.ncbi.nlm.nih.gov/genomes/all/GCA/000/770/565/GCA_000770565.1_ASM77056v1 |
| MSHR1655 | GCA_000756165.1 | ftp://ftp.ncbi.nlm.nih.gov/genomes/all/GCA/000/756/165/GCA_000756165.1_ASM75616v1 |
| MSHR2543 | GCA_000959225.1 | ftp://ftp.ncbi.nlm.nih.gov/genomes/all/GCA/000/959/225/GCA_000959225.1_ASM95922v1 |
| MSHR305 | GCA_000439695.1 | ftp://ftp.ncbi.nlm.nih.gov/genomes/all/GCA/000/439/695/GCA_000439695.1_ASM43969v1 |
| MSHR3763 | GCA_001975145.1 | ftp://ftp.ncbi.nlm.nih.gov/genomes/all/GCA/001/975/145/GCA_001975145.1_ASM197514v1 |
| MSHR4083 | GCA_001975125.1 | ftp://ftp.ncbi.nlm.nih.gov/genomes/all/GCA/001/975/125/GCA_001975125.1_ASM197512v1 |
| MSHR491 | GCA_000959205.1 | ftp://ftp.ncbi.nlm.nih.gov/genomes/all/GCA/000/959/205/GCA_000959205.1_ASM95920v1 |
| MSHR520 | GCA_000583835.1 | ftp://ftp.ncbi.nlm.nih.gov/genomes/all/GCA/000/583/835/GCA_000583835.1_ASM58383v1 |
| MSHR5848 | GCA_000755965.1 | ftp://ftp.ncbi.nlm.nih.gov/genomes/all/GCA/000/755/965/GCA_000755965.1_ASM75596v1 |
| MSHR5855 | GCA_000756065.1 | ftp://ftp.ncbi.nlm.nih.gov/genomes/all/GCA/000/756/065/GCA_000756065.1_ASM75606v1 |
| MSHR5858 | GCA_000755945.1 | ftp://ftp.ncbi.nlm.nih.gov/genomes/all/GCA/000/755/945/GCA_000755945.1_ASM75594v1 |
| MSHR5864 | GCA_001975105.1 | ftp://ftp.ncbi.nlm.nih.gov/genomes/all/GCA/001/975/105/GCA_001975105.1_ASM197510v1 |
| MSHR62 | GCA_000770395.1 | ftp://ftp.ncbi.nlm.nih.gov/genomes/all/GCA/000/770/395/GCA_000770395.1_ASM77039v1 |
| MSHR668 | GCA_000959305.1 | ftp://ftp.ncbi.nlm.nih.gov/genomes/all/GCA/000/959/305/GCA_000959305.1_ASM95930v1 |
| MSHR6755 | GCA_001975085.1 | ftp://ftp.ncbi.nlm.nih.gov/genomes/all/GCA/001/975/085/GCA_001975085.1_ASM197508v1 |
| MSHR7929 | GCA_001975065.1 | ftp://ftp.ncbi.nlm.nih.gov/genomes/all/GCA/001/975/065/GCA_001975065.1_ASM197506v1 |
| MSHR840 | GCA_000959185.1 | ftp://ftp.ncbi.nlm.nih.gov/genomes/all/GCA/000/959/185/GCA_000959185.1_ASM95918v1 |
| NCTC 13178 | GCA_000511895.1 | ftp://ftp.ncbi.nlm.nih.gov/genomes/all/GCA/000/511/895/GCA_000511895.1_ASM51189v1 |
| NCTC 13179 | GCA_000494855.1 | ftp://ftp.ncbi.nlm.nih.gov/genomes/all/GCA/000/494/855/GCA_000494855.1_ASM49485v1 |
| K42 | GCA_000770515.1 | ftp://ftp.ncbi.nlm.nih.gov/genomes/all/GCA/000/770/515/GCA_000770515.1_ASM77051v1 |
| K96243 | GCA_000011545.1 | ftp://ftp.ncbi.nlm.nih.gov/genomes/all/GCA/000/011/545/GCA_000011545.1_ASM1154v1 |
| Burk178-Type1 | GCA_001887555.1 | ftp://ftp.ncbi.nlm.nih.gov/genomes/all/GCA/001/887/555/GCA_001887555.1_ASM188755v1 |
| BPC006 | GCA_000294635.1 | ftp://ftp.ncbi.nlm.nih.gov/genomes/all/GCA/000/294/635/GCA_000294635.1_ASM29463v1 |
