# Supplementary figures and images for: Clinical Burkholderia pseudomallei isolates from north Queensland carry diverse bimABm genes that are associated with central nervous system disease and are phylogenomically distinct from other Australian strains
Source: PLoS Negl Trop Dis. 2022 Jun 14;16(6):e0009482. doi: 10.1371/journal.pntd.0009482 (PMC9236262; doi:10.1371/journal.pntd.0009482)

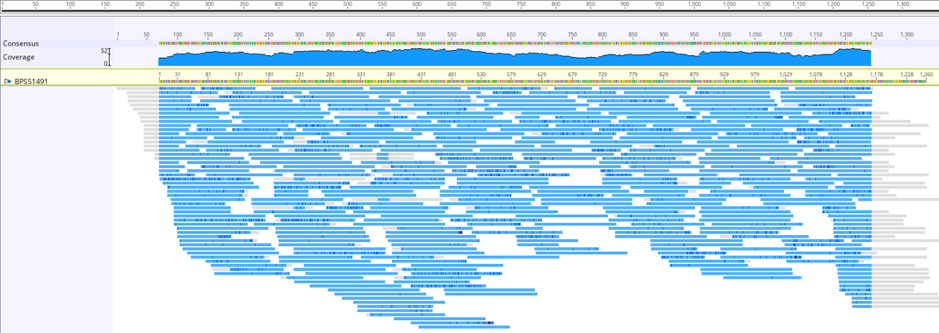

Supplement: S1 Fig — (PNG) [file pntd.0009482.s002.png]
