# Supplementary material for: Clinical Burkholderia pseudomallei isolates from north Queensland carry diverse bimABm genes that are associated with central nervous system disease and are phylogenomically distinct from other Australian strains
Source: PLoS Negl Trop Dis. 2022 Jun 14;16(6):e0009482. doi: 10.1371/journal.pntd.0009482 (PMC9236262; doi:10.1371/journal.pntd.0009482)
Supplement: Attachment — Submitted filename: Response to reviewers commentsv2.docx [file pntd.0009482.s003.docx]

**Reviewer's Responses to Questions (PNTD-D-21-00728)**

**Editorial comments and responses (21^st^ Dec 2021):**

2.) Please ensure that the Title in your manuscript file and the Title provided in your online submission form are the same.

- **These are now the same (the word “north” had been accidentally omitted online**

3.) Please ensure that all author names are spelled correctly in the manuscript file, including the use of a middle name/initials, and any special characters (accents, umlauts, etc.). Please place first/given names before last/family names.  All authors must have a first and last name. We noticed that  David L Paterson is spelled differently between the manuscript file and submission form. Please be aware that correct spelling and formatting of author names in the article file is vital for accurate indexing in PubMed.

- **David Paterson’s name has been amended online - others are correct**

4.) We noticed that you used “data not shown” in the manuscript. We do not allow these references, as the PLOS data access policy requires that all data be either published with the manuscript or made available in a publicly accessible database. Please either remove these references, or amend the supplementary material to include the referenced data.

- **The additional data has bene included as figure S1 in supplementary material**

5.) We do not publish any copyright or trademark symbols that usually accompany proprietary names, eg (R), (C), or TM  (e.g. next to drug or reagent names). Therefore please remove all instances of trademark/copyright symbols throughout the text, including MiniSeqTM on page 6.

- **This has been amended**

6.) In your manuscript, the figure labelled as Figure 1 is uploaded as Figure 3, and vice versa. Please make sure uploaded figures are matching with those referred in the manuscript.

- **Apologies for the error – this has been fixed**

7.) We have noticed that you have uploaded supporting information but you have not included a list of legends.  Please add a full list of legends for all supporting information files (including figures, table and data files) after the references list.

- **This has been added in the manuscript file**

**Reviewer’s comment**

**Results**

-Does the analysis presented match the analysis plan? Are the results clearly and completely presented? Are the figures (Tables, Images) of sufficient quality for clarity?

Reviewer #1: Results are clear and clearly presented.

Reviewer #2: #1) Lines 88-90 and Table 1. Which of the 18 isolates make up the CNS strains? 4 of the strains from CSF and 2 from the brain, but it was not clear where the other 12 come from as listed in Table 1. Also, on line 89, it lists 18 CSF strains and the remaining 57 but that adds up to 75. Line 86 refers to a total of 76 isolates.

Author’s response: A description of the origin of the isolates with documented central nervous system presentations has been included in lines 89-90, we were reluctant to include this in table 1 as some isolates are missing metadata and the numbers could be misinterpreted by readers. The number 57 has been corrected to 58 on line 90.

#2) Figure 2. This reviewer would expand the legend description. It is not clear what I should be focused upon. Do the various colors represent something notable?

Author’s response:

The legend for Figure 2 has been expanded to now read:

**Figure 2.** Alignment of the *BimA_Bm_* proline-rich region identified in Queensland *B. pseudomallei* isolates. Extensive variation of the proline rich region coloured green, is observed between isolates included in this study as well as compared to the Queensland *BimA_Bm_* reference genome MSHR668 located at the top of the alignment.

#3) Line 132. Points out the important amino acid variant N213S for the 5 strains. Should this be included/shown in Fig 2?

#4) Lines 136-139. These results are discussed as being important but not shown.

Author’s response: The mutations described in lines 132-139 have been included in figure 2., as Figure 2a and Figure 2b, respectively. The legend now includes:

Figure 2a. Alignment depicting the four isolates carrying an amino acid variant ΔR11H upstream of the proline rich region of the *BimA_Bm_* gene Figure 2b. Alignment depicting the five isolates carrying an amino acid variant ΔN213S downstream of the proline rich region of the *BimA_Bm_* gene.

--------------------

Conclusions

-Are the conclusions supported by the data presented? Are the limitations of analysis clearly described? Do the authors discuss how these data can be helpful to advance our understanding of the topic under study? Is public health relevance addressed?

Reviewer #1: 259-262 significant amount of genetic diversity in Queensland B.

265	unclear if absence of bimABm could identify low risk of CNS involvement given only half of the patients with CNS infection had the variant

Author’s response: The authors agree it is unknown if the absence of bimABm could indicate low risk of CNS presentation and encourage further exploration into drivers for this type of B. pseudomallei infection.

Reviewer #2: No comments for conclusions.

--------------------

Editorial and Data Presentation Modifications: Use this section for editorial suggestions as well as relatively minor modifications of existing data that would enhance clarity. If the only modifications needed are minor and/or editorial, you may wish to recommend “Minor Revision” or “Accept”.

Reviewer #1: (No Response)

Reviewer #2: #1. Line 37. Should multi-locus sequence types be abbreviated as MLST versus ST? In other places (line 259), the abbreviation is shown as MLST.

Author’s response: MLST and STs has been redefined for clarity in the text on lines 111-113

#2. Table 2. Title refers to bimAps. Should this be bimABp?

Author’s response: Yes, typo has been correct in Table 2 title.

#3. Line 176. Should this be worded: “Twenty isolated carried the “altered” virulence factor”?

Author’s response: Lines 178-179 now read: Of the76 isolates, 20 carried the virulence factor *bimA_Bm_* and only two of the sequences produced from this study were identical (TSV39, TSV25).

--------------------

Summary and General Comments: Use this section to provide overall comments, discuss strengths/weaknesses of the study, novelty, significance, general execution and scholarship. You may also include additional comments for the author, including concerns about dual publication, research ethics, or publication ethics. If requesting major revision, please articulate the new experiments that are needed.

Reviewer #1: This study aimed to identify prevalence of bimABm, which is associated with meningitis/CNS infection, with comparative genomics , has been previously linked to neurological disease in B. pseudomallei infections in patients of the Northern Territory

From the 76 isolates analyzed,the bimABm variant was identified in 20 (26.3%) isolates and 9 (45%) of the isolates with documented central nervous system infection (n=18).

A significant association was noted between isolates carrying the bimABm variant and central nervous system disease (OR 2.8, 95% CI 1.3-6.0, P=0.009) compared with isolates carrying the wildtype bimABp.

This association, however, has been described for over a decade (cit. 7 Stevens Mol Microbiol 2005)

Author’s response: This is correct, the bimABm variant has been known to have actin-based activity for many years (cit. 7 Stevens Mol Microbiol 2005), however it remains significantly understudied if this variation has dispersed in Queensland *B. pseudomallei* and to what extent (cit. 26 Sitthidet J Clin Micro 2008, cit 25 Morris Emerg Infect Dis 2017) .

METHODS: How did the investigators select the control isolates (e.g., Queensland NON-CNS isolates)? Couldn't some apparent differences be introduced between the two groups simply by differences in era of enrollment, location within the geographically large state of Queensland, or even characteristics of the patient (immigrant status, race, gender, age, immunocompromise/diabetic state)?

Author’s response: The other 57 isolates were chosen to be representative of all the isolates in the main collection, including a variety of patient characteristics as mentioned above.

120-126 Half of CNS isolates had bimABm; implying that it is not fully causative.

More interesting is what homologous genes were present in the 9 isolates from CNS disease who did NOT have the bimABm variant.

Further, why did the authors not perform in vitro phenotypic testing to establish phenotypic variation in host actin-mediated mobility between the identified variants?

Alternately they could perform in vivo RNA seq on the bacteria to evaluate potential upstream regulatory differences among the bim ABm variants NOT associated with meningitis; and also among the 9 meningitis-derived isolates that did not manifest the BimABm variant.

Author’s response: This is an excellent idea, however, was beyond the scope of the funding and timeline for this particular project.

151-154 Unsurprising result given proximity of the regions. More interesting are the Thailand derived isolates. Could these have been described amongst recent immigrants

Author’s response: It is very possible this is the case, however with limited metadata we are unable to describe the exact history of these strains.

167-172 Why not phenotypic confirmation of the AMR findings?

Author’s response: This is an excellent idea, however, was beyond the scope of the funding and timeline obtained for this particular project. Data coinciding with AMR was not collected in the same way from different hospitals, therefore existing AMR data was not able to be used either.

259-262 significant amount of genetic diversity in Queensland B.

265	unclear if absence of bimABm could identify low risk of CNS involvement given only half of the patients with CNS infection had the variant

Author’s response: The authors agree it is unknown if the absence of bimABm could indicate low risk of CNS presentation and encourage further exploration into drivers for this type of B. pseudomallei infection.

Reviewer #2: The results are interesting but the manuscript could be tightened in a few areas to better relay the story. Details below.

Author’s response: The authors believe the excellent points made by both reviewers 1 and 2 will result in an improved manuscript.

#1) This reviewer feels more details, distinction, and appropriate references should be provided for bimAbm and bimABp.

a. The description is limited to just one sentence lines 63-67. This section lacks a description of what the difference are in general between these two versions of bimA.

b. Also, it may be a semantics issue, but is it appropriate to call this a mutant? Perhaps more appropriate would be variant or referring to those Bp strains with bimABm as a subset of a population.

c. The sentence starting at line 63 should have an appropriate reference for it.

Author’s response: The paragraph referred to here (lines 63-73) have been significantly revised and now include a more detailed description, differences between the genes and references and now reads:

Central nervous system infections have been associated with the virulence factor bimABp mutant, bimABm, a variant of the wild-type, bimABp [7–9]. These bimA genes encode as intracellular motility factor A, type V effector proteins that utilise host cellular actin. This motility results in the ability to evade the host immune system and invade the central nervous system via inter-cellular host cell migration[10]. The bimABm variant is significantly truncated to that of bimABp and is significantly more similar to bimAMa, of the highly infectious B. mallei, that is fatal in humans if left untreated[11]. Furthermore, the carboxy-terminals of the bimA genes of both species are also significantly similar to the YadA auto-secreted adhesion protein of Yersinia enterocolitica, another highly pathogenic bacteria[10,12,13]. Patients with infections possessing the bimABm gene are suggested to be 14 times more likely to present with neurological disease, while the wild-type variant bimABp is linked to pneumonia[7,14].

#2) As highlighted above, many of the relevant mutations discussed in the results were not shown in the figures or even listed as "data not shown".

Author’s response: This has been corrected as per the above statements, where data has now been included in figure 2 where appropriate.

--------------------
