# Supplementary material for: Clinical Burkholderia pseudomallei isolates from north Queensland carry diverse bimABm genes that are associated with central nervous system disease and are phylogenomically distinct from other Australian strains
Source: PLoS Negl Trop Dis. 2022 Jun 14;16(6):e0009482. doi: 10.1371/journal.pntd.0009482 (PMC9236262; doi:10.1371/journal.pntd.0009482)
Supplement: Attachment — Submitted filename: Response to reviewers comments v4.docx [file pntd.0009482.s004.docx]

Authors Response to Reviewers Comments

Reviewer #3: 1. The overall study design is appropriate and adequately address the hypothesis.
2. My only concern is if the authors have conducted a simple antibiotic susceptibility test on the isolates, particularly those 76 clinical isolates. This simple experiment can perhaps give a clearer correlation to AMR related mutations analysis conducted by the authors.

Authors response: The authors agree with the reviewers’ comments however, this was outside of the scope of this pilot study due to financial constraints.

3. Line 98 - Papua New Guinea

Authors response: This has been corrected to Papua New Guinea

Reviewer #3: 1. The results are clear and well presented.
2. Can the authors prove the correlation between the presence of AMR related mutations with the isolates phenotype (antibiotic susceptibility)?

Authors response: The authors agree this would be great, yet as mentioned above, this was out of our financial scope.

Reviewer #2: Several minor editorial suggestions for the authors.

Lines 35 & 59.  Add abbreviate for CNS as needed.

Authors response: This has been corrected throughout the manuscript.

Line 51.   ....Territory and surrounds.   Is something wrong/ missing with sentence?

Authors response: Sentence now reads: “Studies of melioidosis in Australia are disproportionally inclusive of the Top End of the Northern Territory and its surrounds.”

Line 51.  This geographic....  Should "area" be added?

Authors response: “area” has been added.

Line 57.  Something missing from the start of this sentence?  "Infections cause the disease meliodosis....

Authors response: The sentence has been revised to read: “The disease melioidosis, caused by *B. pseudomallei* infection, can present with an extensive range of disease manifestations including, but not limited to, pneumonia, sepsis, and skin and soft tissue abscesses[4].”

Line 68.  bimAMa- is this supposed to be bimABm?

Authors response: This sentence is correct and is referring to the bimA variant of the species *B. mallei*.

Line 74.  I would recommend saying which gene we are speaking of bimA, bimABp, or bimABm?

Authors response: The sentence now begins with “The *bimA_Bp_* gene”.

Line 151.  What is "ace"?

Authors response: The sentence now reads: “Novel allele variants were identified across six of the alleles, with the allele *ace* the only one without any variants (n=12).”

line 186 &211.  This is gene, correct?  Should be "b".

Authors response: The authors are unsure as to what is being asked here but have checked the surrounding sentences for any errors in reference to genes and all seem to be correct.

Reviewer #4:

Line 24: with antibiotic treatment?

Authors response: The sentence now reads: “In Australia, the mortality rate remains high at approximately 10%, despite curative antibiotic treatment being available.”

Line 74: precise the version of the gene

Authors response: This has been corrected as above.

Line 223-224: could be interesting to study this with mutants and wildtype infections

Authors response: We authors agree that this type of study could be extended in the future to include mutant vs wildtype comparisons.

Line 238: are the alleles related to virulence genes?

Authors response: The *bimA* genes were the only virulence genes studied. Multi locus sequence type alleles are not related to or reflect virulence genes in B. pseudomallei.

Table 2: problem in the percentage column

Authors response: We see how the percentage columns could have been misleading to the readers and have adjusted accordingly for clarity.

Figure 2: legend title ‘Alignment of…’

Authors response: A space has been added on line 315 in the legend title.
